# A new anthropometric index for body fat estimation in patients with severe obesity

**DOI:** 10.1186/s40608-018-0202-8

**Published:** 2018-10-01

**Authors:** Giliane Belarmino, Raquel S. Torrinhas, Priscila Sala, Lilian M. Horie, Lucas Damiani, Natalia C. Lopes, Steven B. Heymsfield, Dan L. Waitzberg

**Affiliations:** 1 Department of Gastroenterology, Surgical Division, LIM 35, Faculdade de Medicina da Universidade de São Paulo, Av. Dr. Arnaldo, 455, 2° andar, sala 2208, São Paulo, Cerqueira César CEP: 01246-903 Brazil; 2 Research Institute – Hospital do Coração de São Paulo, São Paulo, Brazil; 3 Pennington Biomedical Research Center, Baton Rouge, LA USA

**Keywords:** Severe obesity, Body fat, Body adiposity index, Air displacement plethysmography

## Abstract

**Background:**

Body mass index (BMI) has been used to assess body adiposity, but it cannot adequately reflect body fat (BF) amount. The body adiposity index (BAI) has been shown a better performance than BMI for this purpose, but it can be inaccurate to estimate BF under extreme amounts of fat. Here, we propose a new anthropometric index, the Belarmino–Waitzberg (BeW) index, for specific estimation of BF in severely obese patients.

**Methods:**

In 144 adult patients with severe obesity, BF was estimated by air displacement plethysmography (ADP), as the reference method, along with the follow anthropometric measurements: height, abdominal circumference (AC), hip circumference (HC), weight, BMI (weight/ height^2^) and BAI ([HC(cm) / height (m)^1.5^) − 18] × 100). Patients were proportionately distributed into two distinct databases, the building model database (BMD) and the validation model database (VMD), which were applied to develop and validate the BeW index, respectively. The BeW index was tested for gender and ethnicity adjustment as independent variables. The agreement of BF% values obtained by the new index and by BAI with ADP was also assessed.

**Results:**

The BF% was 52.05 ± 5.42 for ADP and 59.11 ± 5.95 for the BeW index (all results are expressed as the mean ± standard deviation). A positive Pearson correlation (*r* = 0.74), a good accuracy (Cb = 0.94), and a positive Lin’s concordance correlation (CCC = 0.70) were observed between the two groups. The 95% limits of individual agreement between the BeW index and ADP were 6.8 to 7.9%, compared to − 7.5 to 14.8% between the BAI and ADP. The new index, called the Belarmino–Waitzberg (BeW) index, showed an improvement of 2.1% for the R^2^ value and a significant gender effect, therefore resulting in two different indexes for females and males, as follows: Female BeW = − 48.8 + 0.087 × AC(cm) + 1.147 × HC(cm) - 0.003 × HC(cm)2 and Male BeW = − 48.8 + 0.087 × AC(cm) + 1.147 × HC(cm) - 0.003 × HC(cm)2–7.195.

**Conclusions:**

The new BeW index showed a good performance for BF estimation in patients with severe obesity and can be superior to the BAI for this purpose.

## Background

The obesity epidemic is now a major public health concern due its impact on health and the economy. Characterized by abnormal body fat (BF) accumulation, obesity is linked with several debilitating diseases and an increased mortality [1]. An accurate diagnosis of obesity can guide clinical management and treatments.

The body mass index (BMI) has been widely applied to identify and classify obesity. However, the BMI cannot fully reflect the amount and distribution of BF or clearly distinguish the fat-free mass from fat mass compartments. Particularly, the BMI performance in severely obese patients is impaired by the large amount of subcutaneous adipose tissue and edema [2, 3].

BF assessment is more precise in obese subjects when more specific methods for body composition evaluation are used. Air displacement plethysmography (ADP) is a scientific validated reference method to evaluate body composition in obese subjects [4]. The method uses air displacement after patient entry in the ADP device to assess BF, by applying the Boyle’s Law [5]. ADP has several advantages over other reference methods for BF assessment, including a quick noninvasive measurement process [5]. The main limitation in the routine application of ADP or other similar available tools for BF assessment is its high cost and practical complexity. Therefore, other alternative methods for BF assessment have been proposed.

In particular, the body adiposity index (BAI) has been found to be more sensitive to identify and classify obesity than the BMI, compared to dual-energy X-ray absorptiometry (DXA), as the reference method [6]. The BAI provides a practical advantage because it uses a simple equation for calculation that includes only hip circumference (HC) and body height. Furthermore, the BAI was developed from a database composed of adult Mexican-American men and further successfully validated in adult African-American men and women, suggesting that it could be applied universally without adjustments for gender or race.

Nevertheless, studies applying the BAI have shown its limited performance for BF estimation in populations with extreme amounts of fat (very low or very high) [7–9]. In particular, we have recently shown that the BAI poorly estimates BF in severely obese patients. Therefore, the aim of the current study was to design a new index to estimate BF in patients with severe obesity using simple and accessible anthropometric measurements.

## Methods

### Study protocol and design

Our study protocol was designed to develop a new anthropometric index for BF estimation in severely obese patients. For this purpose, our obese patient sample was composed of two proportional and distinct databases: the building model database (BMD), composed of the same 72 patients for whom we previously found a low performance of the BAI in estimating BF [10]; and the validation model database (VMD), composed of new 72 paired obese patients. The BMD was applied to develop the new index from anthropometric data potentially predictive of BF, using the BF% obtained by ADP as reference standard. The VMD was applied to validate the new index by assessing its performance in estimating BF in a similar population. For this purpose, the correlation and agreement between the BF values estimated by the new index with the BF values estimated by ADP were assessed. Furthermore, the agreement between the BF values estimated by BAI with the BF values estimated by ADP also was assessed to identify the performance of BAI comparative to the new index. For all the analyses, each patient was evaluated on the same day, in the morning, and after a 4-h overnight fast. Patients were instructed not to smoke or to drink alcohol during the 24 h prior to measurements, which were performed by the same trained technician for all enrolled patients.

### Patients

Sample size calculations were based on the development of a model with nine possible predictors, with an effect size of at least 0.15 and considering an alpha value of 5% and a power of 95% [11]. A total of 144 severely obese Brazilian patients (108 female, 36 male) aged 18–55 years old, who were candidates for bariatric surgery and each of whom had a BMI ≥ 30 kg/m^2^ (range: 30–62 kg/m^2^), were recruited from the Digestive Tract Surgery Service at the Hospital das Clinicas - University of São Paulo School of Medicine, São Paulo, Brazil. The exclusion criteria were neurologic or psychiatric conditions; substance abuse; lactating or pregnant women; HIV-positive or cancer patients; clinically detectable edema; physical amputations; and chronic or acute diseases of the liver, lung, kidney, or heart. All study procedures were performed according to the ethical standards of the World Medical Association’s Declaration of Helsinki. These and both datasets (building and validation) were approved by the institutional ethics review board – CAPPesq (1069/05 and 1011/09). Written informed consent was obtained from each patient prior to participation.

### Anthropometric data

Each anthropometric data corresponds to an average of 3 sequentially repeated measures, which were performed as previously described elsewhere [10]. Briefly, body weight (kg, minimal variation of 10 g) was measured by using the weekly-calibrated body weight scale of the ADP system (Bod Pod body composition system, Life Measurement Instruments, Concord, CA, USA), with the patient standing in the center of the scale platform, barefoot, and wearing only underwear. Body height (cm) was obtained with a stadiometer (Sanny, São Paulo, Brazil), with the patient standing, barefoot with the heels together, back upright, and arms stretched next to the body. The abdominal circumference (AC) was measured using an inelastic metrical tape at the trunk midway between the lower costal margin (bottom of the lowest rib) and the iliac crest (top of the pelvic bone), with the patient standing with his/her feet 25–30 cm apart. The measurement was taken by fitting the tape snugly, without compressing the underlying soft tissue. Circumference was measured to the nearest 0.5 cm, at the end of a normal expiration [12]. The HC (cm) was measured by positioning a measuring tape in the horizontal plane at the greatest circumference of the buttocks [6, 13]. In addition, the BMI was calculated as body weight (kg) / [height (m)]^2^ and classified according to the World Health Organization scoring system [14, 15].

### BF estimation by the BAI

The following equation was used to estimate BF by the BAI: BF% BAI = [(HC(cm) / height (m)^1.5^) − 18] × 100^6^.

### BF estimation by ADP

Using the Bod Pod, ADP was performed to estimate the total BF. In the ADP method, the inverse relationship between pressure and volume proposed by Boyle (P1 × V1 = P2 × V2) was used to determine the body volume. Skin surface area artefact (*SAA*) also was calculated by the BOD POD software to allow changes in air temperature close to the subject’s skin. Body volume (BV) and body density (BD) were then calculated as V (L) = *BVraw* – *SAA* (L) + *40*% *TGV* (L) and BF% was then calculated using Siri’s equation: BF% = (4.95/D – 4.5) × 100, where D = density. All measurements and calculations were automatically performed by the system software, and they are based on air volume and pressure variations inside the Bod Pod chamber when occupied and not occupied by the patient [12, 16]. During ADP evaluations, the patients wore only underwear and a cap to keep their hair fastened, and they remained in a sitting position inside the chamber [12]. Metallic objects, such as earrings, rings, chains, and body piercings, were not allowed.

### New index design

Values of BF% obtained by ADP from the BMD were correlated with anthropometric data. Variables with a significant concordance with the BF% values provided by ADP were included in the initial index model and tested for the influence of gender and race by polynomial regression. The Akaike criterion was applied to select the variables to be used in the final index model, and a backward linear regression was applied to develop the specific BF prediction equation.

### New index test for validation and performance

The agreement between the BF values from the ADP system with the two new BF equations and BAI were assessed according to the Pearson correlation, (r), accuracy (Cb), Lin’s concordance correlation coefficient (CCC) and the Bland–Altman plot.

### Statistical analysis

In addition to the statistical analysis described above for the development of validation of the new equation, descriptive data were compared by the Student’s unpaired t- test or the Mann-Whitney U-test, when appropriate. All statistical analyses were performed using the R software package (version 3.1.0, R Development Core Team, 2014). The results are expressed as the mean ± standard deviation. Statistical significance was set at *p* <  0.05 for all tests.

## Results

### Descriptive data

Table 1 provides the baseline demographic, body composition, and anthropometric data of the studied obese patients, divided in into the MBD (*n* = 72) and MVD (n = 72) as well as the total (*n* = 144). From the entire sample, most of the patients were women (70%), the BMI ranged from 34.40 to 62.98 kg/m^2^, with 92% of patients having BMI > 40 kg / m^2^; age ranged from 18 to 62 years old; and the mean BF% estimates measured by ADP was 52.05 ± 5.42%.

**Table 1:** Demographic, body composition, and anthropometric data of severely obese patient samples

Variable	MBD	MVD	Total (*n* = 144)	*P* value
Gender (female)	53/72 (73.6%)	55/72 (76.4%)	108/144 (75.0%)	0.847
Gender (male)	19/72 (26.4%)	17/72 (23.6%)	36/144 (25.0%)	0.281
Race (white)	46/72 (63.9%)	53/72 (73.6%)	99/144 (68.8%)	
Race (black/brown)	26/72 (36.1%)	26/72 (36.1%)	45/144 (31.2%)	0.877
Age (years)	42.58 ± 12.32	42.88 ± 10.07	42.73 ± 11.21	0.318
Body weight (kg)	127.68 ± 27.5	123.33 ± 24.53	125.51 ± 26.06	0.386
Height (m)	1.63 ± 0.1	1.62 ± 0.1	1.63 ± 0.1	0.532
BMI (kg/m^2^)	47.45 ± 6.75	46.77 ± 6.23	47.11 ± 6.48	0.719
AC (cm)	137.46 ± 17.38	136.49 ± 14.83	136.97 ± 16.11	0.728
HC (cm)	135.21 ± 13.83	136.02 ± 13.97	135.61 ± 13.86	0.565
Waist-hip ratio	1.02 ± 0.11	1.01 ± 0.11	1.01 ± 0.11	0.847
ADP BF%	52.14 ± 5.4	51.96 ± 5.47	52.05 ± 5.42	0.300

Data were obtained from 144 patients and are expressed as the mean ± standard deviation. *MBD* model building database, *MVD* model validation database, *BMI* body mass index, *AC* abdominal circumference, *HC* hip circumference, *ADP BF%* values of body fat percentage estimated by air displacement plethysmography

### New anthropometric index design

**Fig. 1:**
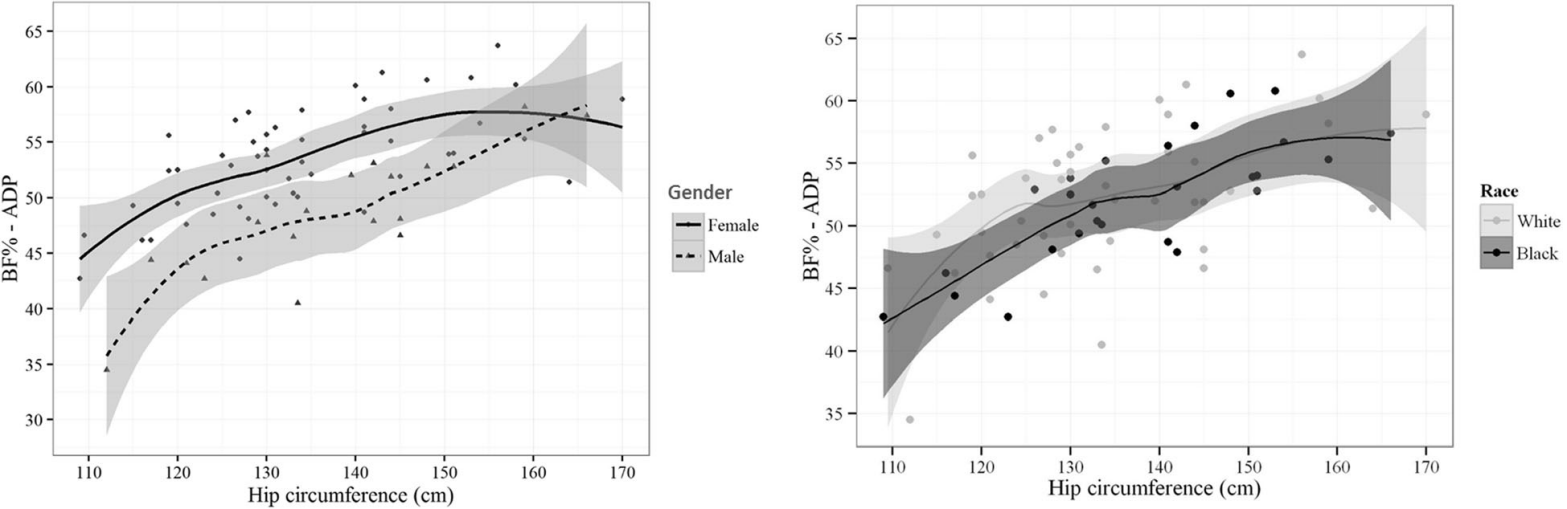
Dispersion graphs of the body fat values (%) estimated by air displacement plethysmography with hip circumference, gender, and race in the model building database. Data were obtained from 72 severely obese patients. The estimated curves were generated by the local polynomial regression method to best describe the data behavior. Note that for gender, the ratio of values of body fat (%) estimated by air displacement plethysmography is not linear, and a curve trend of second order (quadratic) seems to be a good fit to the data. However, for race, there was no effect

Data of BF% estimated by ADP significantly correlated with the following individual anthropometric data: body weight, AC, waist-hip ratio (negative correlation), and HC (*p* ≤ 0.05; Table 2). HC was the variable with the higher significant correlation with BF% estimated by ADP (*p* <  0001) and then was tested for gender and race adjustments. HC shows to be significantly influenced by gender, by displaying a nonlinear dispersion with BF% estimated by ADP (Fig. 1). Therefore, the initial model aimed to join two factors: the highly significant nonlinearity of HC (compared to BF%) and the inclusion of body weight and/or AC as potential additional predictive variables of BF adjusted by gender and race. The quadratic effect of the interaction between HC, body weight, and AC data with gender and race showed that only the HC (quadratic) presented significant results (Table 3). Therefore, by using the Akaike criteria, the new anthropometric index was simplified to the factors shown in Table 4. The new index, called the Belarmino–Waitzberg (BeW) index, showed an improvement of 2.1% for the R^2^ value and a significant gender effect, therefore resulting in two different indexes for females and males, as follows:

$$ \mathrm{Female}\;\mathrm{BeW}=-48.8+0.087\times \mathrm{AC}\left(\mathrm{cm}\right)+1.147\times \mathrm{HC}\left(\mathrm{cm}\right)-0.003\times \mathrm{HC}{\left(\mathrm{cm}\right)}^2 $$

$$ \mathrm{Male}\;\mathrm{BeW}=-48.8+0.087\times \mathrm{AC}\left(\mathrm{cm}\right)+1.147\times \mathrm{HC}\left(\mathrm{cm}\right)-0.003\times \mathrm{HC}{\left(\mathrm{cm}\right)}^2-7.195 $$

**Table 2:** Pearson’s correlation coefficient of body fat values (%) estimated by air displacement plethysmography and anthropometric variables from building database

Variable	r	95% CI	*p* Value*
Body weight (kg)	0.26	0.03–0.46	0.028
Height (m)	−0.16	− 0.37–0.08	0.19
AC (cm)	0.27	0.04–0.47	0.022
HC (cm)	0.59	0.41–0.72	< 0.001
Waist-hip ratio	−0.24	−0.45–-0.01	0.042

Data were obtained from 72 obese patients. *AC* abdominal circumference, *HC* hip circumference

**Table 3:** Coefficients estimated for the initial prediction model for values of body fat (%) estimated by air displacement plethysmography in the model building database

Coefficient	Estimate	Standard Error	t Value	*p* Value
Intercept	−73.52	47.35	−1.55	0.126
AC (cm)	0.07	0.07	1.07	0.288
Body weight (kg)	0.01	0.07	0.20	0.845
HC (cm)	1.55	0.69	2.23	0.030
(HC (cm))^2^	−0.01	0.00	−2.00	0.051
Sex – Male	41.44	88.00	0.47	0.640
Race – brown/black	−2.44	78.92	−0.03	0.976
AC (cm): male	0.13	0.14	0.91	0.367
Body weight (kg): male	−0.14	0.12	−1.13	0.262
HC (cm): sex – male	−0.93	1.30	−0.72	0.477
(HC (cm))^2^: sex – male	0.00	0.00	0.92	0.362
AC (cm): race – brown/black	−0.12	0.16	−0.75	0.459
Body weight (kg): race – brown/black	0.09	0.11	0.80	0.426
HC (cm): race – brown/black	0.14	1.18	0.12	0.904
(HC (cm))^2^: race – brown/black	0.00	0.00	−0.17	0.868
R^2^ = 57.3%				

Data were obtained from 72 obese patients. *AC* abdominal circumference, *HC* hip circumference

**Table 4:** Estimated coefficients of the final prediction model for values of body fat (%) estimated by air displacement plethysmography in the model building database

Coefficient	Estimate	Standard Error	t Value	*p* Value
Intercept	−48.805	33.124	−1.473	0.145
Abdominal circumference (cm)	0.087	0.035	2.439	0.017
Hip circumference (cm)	1.147	0.486	2.358	0.021
(Hip circumference (cm))^2^	−0.003	0.002	−1.981	0.052
Sex – Male	−7.195	1.123	−6.405	< 0.001
R^2^ = 59.4%				

Data were obtained from 72 obese patients

### New anthropometric index validation and performance

**Fig. 2:**
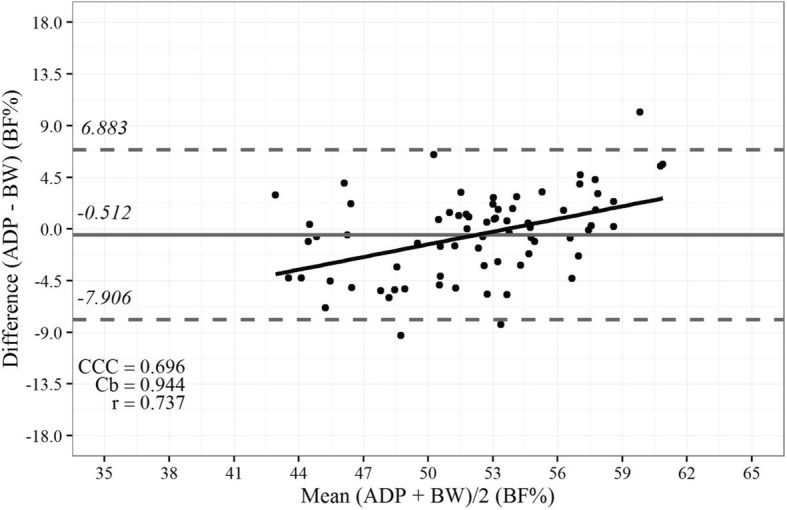
Bland-Altman plot showing limits of agreement between the values of body fat (%) estimated by air displacement plethysmography (ADP) vs. those estimated by the new Belarmino–Waitzberg (BeW) index in the validated model sample. Data were obtained from 72 severely obese patients. Bold continuous lines indicate the observed average agreement. Continuous lines indicate the line of perfect average agreement. Dashed lines indicate 95% limits of agreement. Lin’s concordance correlation coefficient (CCC) is shown

**Fig. 3:**
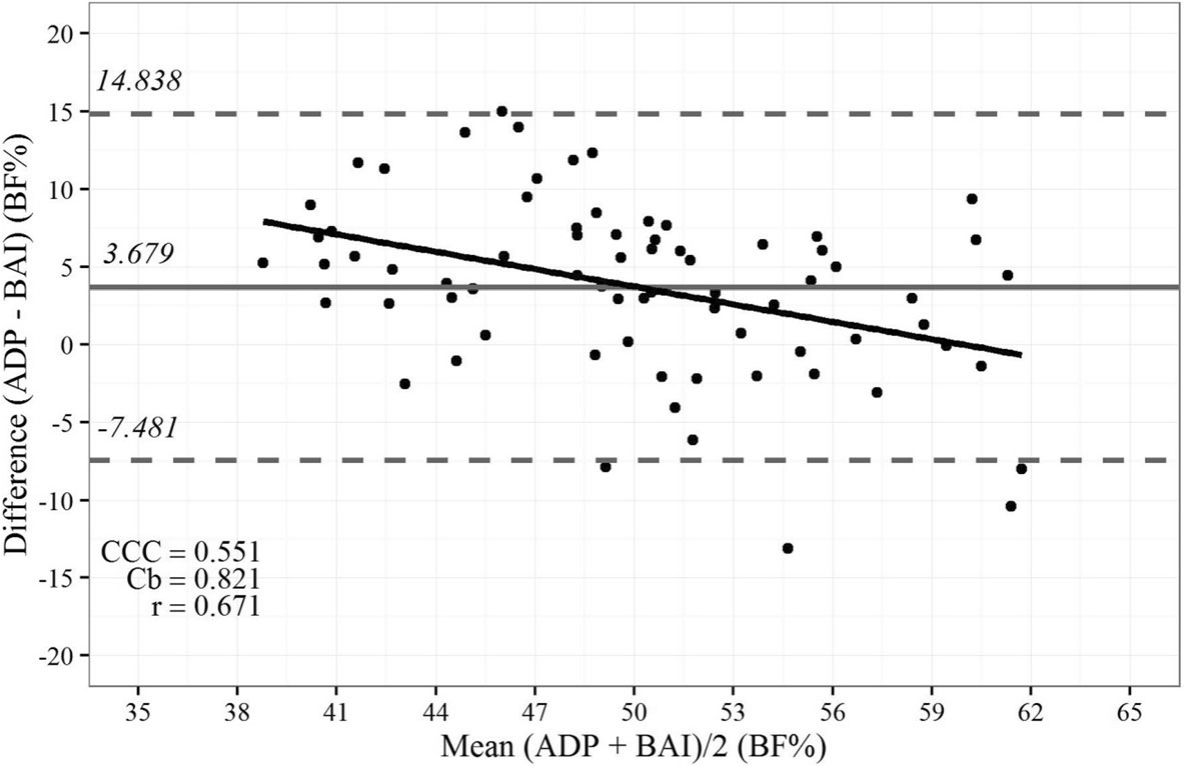
Bland-Altman plot showing limits of agreement between the values of body fat (%) estimated by air displacement plethysmography (ADP) vs. those estimated by the body adiposity index (BAI) in the validated model sample. Data were obtained from 72 severely obese patients. Bold continuous lines indicate the observed average agreement. Continuous lines indicate the line of perfect average agreement. Dashed lines indicate 95% limits of agreement. Lin’s concordance correlation coefficient (CCC) is shown

The new equations of the BeW index were tested for the MVD and showed a good correlation, accuracy, and CCC (*r* = 0.74; Cb = 0.94; and CCC = 0.70, respectively) with BF% estimated by ADP (Fig. 2). Although in a less extend, BF% estimated by ADP also showed a good correlation, accuracy, and CCC with BAI (*r* = 0.67, Cb = 0.82; and CCC = 0.55, Fig. 3); however, the BeW index provided lower limits (6.8 to 7.9%, Fig. 2) of agreement with BF% estimated by ADP than those obtained from the BAI (− 7.5 to 14.8%, Fig. 3). Therefore, the BF% values estimated by the BAI are, on average, 3.7% higher than those estimated by ADP, compared to 0.4% for the new index.

## Discussion

Excessive BF is associated with the occurrence of clinical complications that compromise the quality of life and survival of individuals, such as diabetes, hypertension, cardiovascular disease, musculoskeletal disorders, and cancer [17]. Clinical assessments of BF% in severely obese patients are challenged by the high cost of available methods and the lack of accuracy that some of these methods provide under the elevated fat, total and extracellular water, and change in electrolyte concentration exhibited by severely obese patients [3]. Aiming to solve this issue, we proposed a new equation involving simple anthropometric data to estimate BF% specifically in severely obese patients.

Our study was inspired by the BAI developed by Bergman and colleagues [6]. This tool was designed for BF% assessment, and its most relevant aspects are the simplicity with which it is clinically applied and its higher performance than the BMI to identify and classify obesity. However, this tool has shown a low accuracy at estimating BF% in several populations of severely obese patients [9, 10, 18–21]. These observations highlight that the BAI may not exceed the BMI limitations when applied to assess body composition in severely obese patients [22].

For instance, Geliebter and colleagues compared the BAI and BMI performances in estimating the BF% of severely obese women, using ADP, DXA, and body impedance analysis (BIA) as reference methods [20]. They found that body adiposity estimated by the BMI has a higher correlation with that estimated by ADP, bioelectrical impedance analysis (BIA), or DXA than by BAI. The authors concluded that the BAI seems to be a reasonable method to evaluate BF%, but it should not be used for this purpose in severely obese patients.

The reasons that the BAI is a poor indicator of BF% in severely obese patients may be associated with the use of AC without applying distinct cutoff points for men and women. Obesity is characterized by a distinct fat accumulation between genders, the so-called android and gynoid obesities [23]. Men have a greater fat accumulation in the abdominal area, while in women this occurs in the hip. This feature allows differentiating the body compositions of men and women and may be more pronounced in severely obese patients due the marked fat accumulation.

In fact, by not considering specific male and female cutoffs, the BAI may become a weak tool for BF% evaluation by gender. In a study of the general Mexican-American population, Lichtash and colleagues found that the BAI was a better indicator to estimate body adiposity than the BMI, compared to the reference values of DXA [24]. However, when the authors stratified the population by gender, the BAI had a lower performance than BMI for the same purpose. Notably, when reviewing the limitations for the BAI in assessing the BF% in severely obese patients, Bernhard and colleagues found that significant errors of this variable estimation by BAI were determined by specific factors that included waist-hip ratio ≥ 1.05–5%, gender, and obesity grade [19].

In our study, a poor performance of the BAI in estimating the BF% in severely obese patients was confirmed. For both the MBD and MVD, the BAI underestimated the BF% compared to ADP. Furthermore, we observed that the BF% and HC were not linear between genders. This finding supports a potential need for the design of specific indexes for men and women. In fact, when we designed the new index stratified by gender, we observed better limits of individual agreement of ADP with the BeW index than with the BAI.

The experimental protocol of this study has both strengths and weaknesses. Our sample enrolled mostly severely obese patients (BMI = 47.11 ± 6.48), and this feature was decisive when choosing the reference method for BF% assessment. ADP is a noninvasive method with a high accuracy for BF% estimation from the body density, which has been validated in severely obese patients [2, 4, 25–30]. Several studies have used DXA as a reference method for body composition [6, 9, 20, 24, 31]. However, DXA devices have limited support for weight and width, which can prevent the proper function of the whole-body scanner for severely obese patients [3]. By using ADP as the reference method, we were able to assess the performance of the new index with reliable parameters. On the other hand, our sample was relatively small compared to most available related studies. Nevertheless, it had an effect size of at least 0.15, considering an alpha value of 5% and a power of 95%.

## Conclusion

In summary, our study supports that the BAI may not be reliable for BF% estimation in severely obese patients. In the present study, the new BeW index developed to assess BF% in severely obese patients showed a better performance than the BAI and followed a design that ensures its easy application. Therefore, replacement of the BAI with the new BeW index should be considered when assessing the adiposity level in patients with severe obesity.

## Abbreviations

AC: Abdominal circumference
ADP: Air displacement plethysmography
BAI: Body adiposity index
BeW: Belarmino–Waitzberg
BF: Body fat
BMD: Building model database
BMI: Body mass index
Cb: Accuracy
CCC: Lin’s concordance correlation
DXA: Dual-energy X-ray absorptiometry
HC: Hip circumference
r: Pearson correlation
VMD: validation model database
